# Added value of the EUSOBI diffusion levels in breast MRI

**DOI:** 10.1007/s00330-023-10418-4

**Published:** 2023-11-07

**Authors:** Chiara Zuiani, Iris Mansutti, Guido Caronia, Anna Linda, Viviana Londero, Rossano Girometti

**Affiliations:** 1 Institute of Radiology, Department of Medicine, University of Udine, University Hospital S. Maria Della Misericordia, Azienda Sanitaria Universitaria Friuli Centrale (ASUFC), Udine, Italy; 2 Institute of Radiology, University Hospital S. Maria Della Misericordia, Azienda Sanitaria Universitaria Friuli Centrale (ASUFC), Udine, Italy

**Keywords:** Breast neoplasms, Diffusion magnetic resonance imaging, Diagnosis

## Abstract

**Objectives:**

To investigate whether using the diffusion levels (DLs) proposed by the European Society of Breast Imaging (EUSOBI) improves the diagnostic accuracy of breast MRI.

**Materials and methods:**

This retrospective study included 145 women who, between September 2019 and June 2020, underwent breast 1.5-T MRI with DWI. Reader 1 and reader 2 (R1-R2) independently assessed breast lesions using the BI-RADS on dynamic contrast-enhanced imaging and T2-weighted imaging. DWI was subsequently disclosed, allowing readers able to measure lesions ADC and subjectively express the overall risk of malignancy on a 1–5 Likert scale. ADCs were interpreted as a range of values corresponding to the EUSOBI DLs. The analysis evaluated the inter-reader agreement in measuring ADC and DLs, the per-DL malignancy rate, and accuracy for malignancy using ROC analysis against histological examination or a 3-year follow-up.

**Results:**

Lesions were malignant and showed non-mass enhancement in 67.7% and 76.1% of cases, respectively. ADC was measurable in 63.2%/66.7% of lesions (R1/R2), with a minimal discrepancy on Bland–Altman analysis and 0.948 (95%CI 0.925–0.965)/0.989 (95%CI 0.988–0.991) intraclass correlation coefficient in measuring ADC/DLs. The malignancy rate (R1/R2) increased from 0.5/0.5% (“very high” DL) to 96.0/96.8% (“very low” DL), as expected. Likert categorization showed larger areas under the curve than the BI-RADS for both R1 (0.91 versus 0.87; *p* = 0.0208) and R2 (0.91 versus 0.89; *p* = 0.1171), with improved specificity (81.5% versus 78.5% for R1 and 84.4% versus 81.2% for R2).

**Conclusion:**

Though ADC was not measurable in about one-third of lesions, DLs were categorized with excellent inter-reader agreement, improving the specificity for malignancy.

**Clinical relevance statement:**

DLs proposed by the EUSOBI are a reproducible tool to interpret the ADC of breast lesions and, in turn, to improve the specificity of breast MRI and reduce unnecessary breast biopsies.

**Key Points:**

*• The European Society of Breast Imaging proposed diffusion levels for the interpretation of the apparent diffusion coefficient in diffusion-weighted imaging of the breast.*

*• Adding diffusion levels to the interpretation of magnetic resonance imaging improved the diagnostic accuracy for breast cancer, especially in terms of specificity.*

*• Diffusion levels can favor a more widespread and standardized use of diffusion-weighted imaging of the breast.*

**Supplementary Information:**

The online version contains supplementary material available at 10.1007/s00330-023-10418-4.

## Introduction

Dynamic contrast-enhanced imaging (DCE) represents the core of magnetic resonance imaging (MRI) of the breast, showing 81–100% sensitivity for malignancy [1]. Diffusion-weighted imaging (DWI) has progressively emerged as a valuable tool to complement DCE to improve the characterization of breast lesions [2–4] and, in turn, decrease the number of false positives of breast MRI and unnecessary biopsy recommendations [5]. Although DWI is widely used by most experienced breast radiologists [5], its routine implementation in breast MRI protocols has not been fully established. This is exemplified by the fact that the Breast Imaging Reporting and Data System (BI-RADS) does not provide specific criteria for interpreting DWI [6].

A major factor limiting the widespread adoption of DWI is the difficulty in standardizing the interpretation of the apparent diffusion coefficient (ADC), i.e., the DWI-derived metric quantitatively expressing the random motion of water molecules within normal and diseased breast tissues [7]. While ADC values are typically lower in malignant tumors than benign tumors [8], it is difficult to establish definite ADC thresholds for malignancy or different tumor types, mainly because of the variability in acquisition parameters and analysis methods across different centers and vendors [9]. In order to promote expanded and reproducible use and overcome the problem of defining a threshold for malignancy, a recent consensus and mission statement from the DWI working group of the European Society of Breast Imaging (EUSOBI) proposed a standard for technical acquisition of breast DWI and a classification of the diffusion level (DL) of breast lesions for interpretation [7]. Five different ranges of ADC values derived from a previous metanalysis [10] were assumed to correspond to as many DLs (i.e., very low, low, intermediate, and high) and were, in turn, supposed to be typical of different types of benign and malignant breast lesions [7].

In the EUSOBI proposal, DLs are meant to objectively describe DWI-related information rather than a stand-alone diagnostic tool. DLs should always be used in conjunction with all other MRI data for the purpose of lesion characterization. To our knowledge, no previous studies validated the DLs proposed by EUSOBI as a diagnostic tool. While Bickel et al [11] recently proposed DLs derived from a large multicentric dataset of ADC values rather than literature data, they did not evaluate their diagnostic performance in combination with DCE-based MRI. It is then still unclear how ADC categorization can influence lesion characterization when integrated with BI-RADS-based interpretation and whether the expected role of improving specificity can be achieved.

This study aimed to investigate the malignancy rate on a per-DL basis and the impact on the diagnostic performance of adding the information related to DLs to BI-RADS categorization.

## Materials and methods

### Study population and standard of reference

The referring Institutional Review Board approved this study. The acquisition of written informed consent from patients was waived because of the retrospective design.

**Fig. 1:**
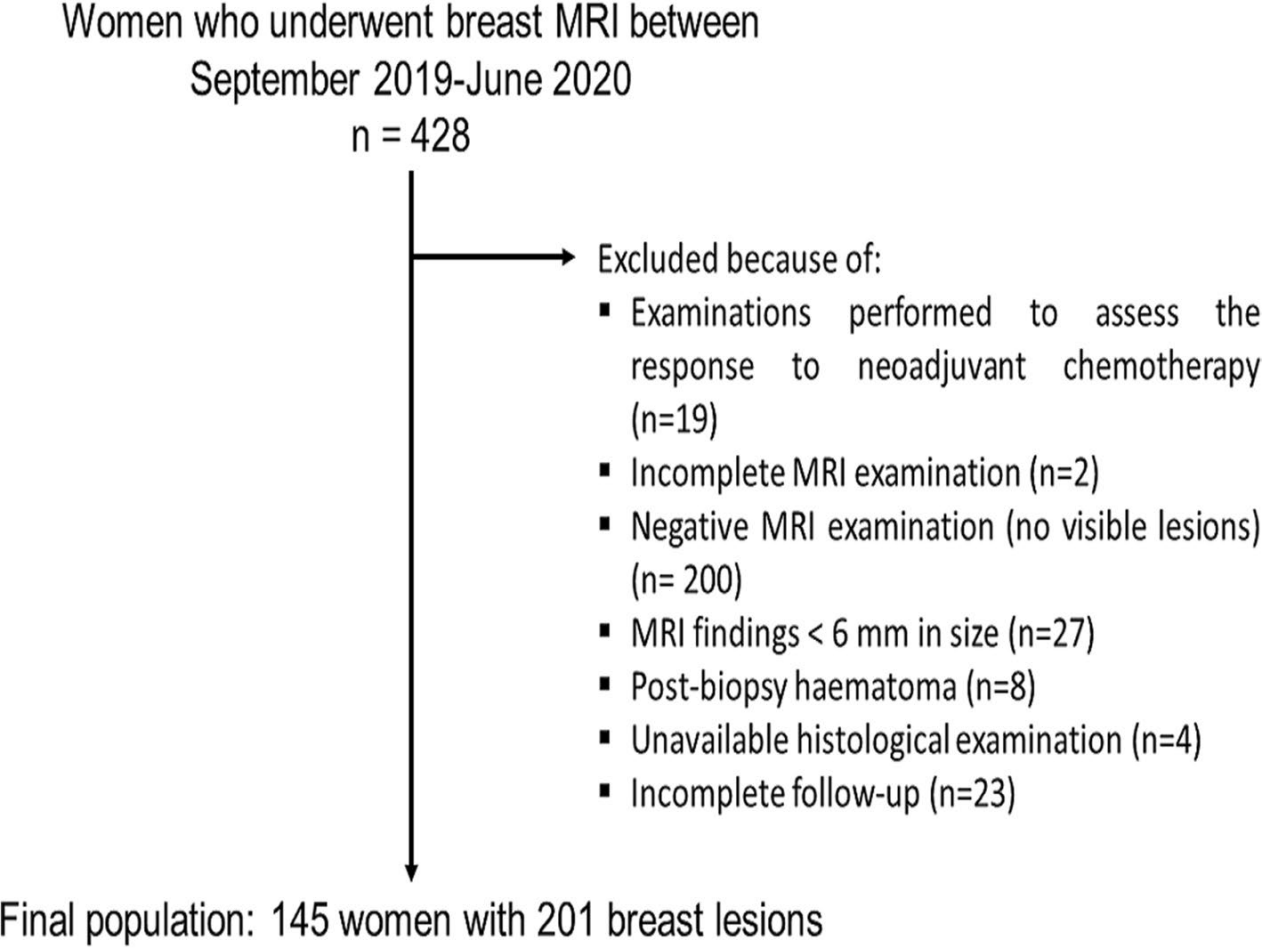
Study flowchart. MRI, magnetic resonance imaging

We identified all the women who underwent breast MRI between September 2019 and June 2020 in our Institution. In line with our activity as a tertiary referral center [12], the examination was performed for various indications, represented mainly by preoperative assessment and problem-solving imaging after inconclusive first-line imaging. After excluding 283 patients (Fig. 1), we finally included 145 women (mean age 53 years, range 21–81 years) showing 201 breast lesions (136 malignant and 65 benign). Factors supposed to limit the applicability of DWI were part of the exclusion criteria, i.e., lesion size < 6 mm or masking effect by post-biopsy hematoma [7].

Final diagnosis was available through biopsy in 39/201 lesions and pathological examination after surgery in 142/201 lesions. Breast biopsy was usually performed before MRI in the case of preoperative examinations and before or after MRI, depending on the indication of the examination and clinical scenario. On a per-patient basis, surgery included bilateral mastectomy (*n* = 2), unilateral mastectomy (*n* = 60), quadrantectomy (*n* = 31), lumpectomy (*n* = 6), surgical excisional biopsy (*n* = 8), and local excision of recurrent breast cancer after mastectomy (*n* = 2). Biopsy was performed under ultrasound (*n* = 36), tomosynthesis (*n* = 2), and MRI (*n* = 1) guidance by one of three breast radiologists with 10–20 years of experience, respectively. One of three pathologists with 6–26 years of experience evaluated biopsy and surgical specimens per the guidelines of the College of American Pathologists since 2020, using the version available when the analysis was performed [13, 14].

In the case of 20 remaining lesions categorized as benign and with no histopathological analysis, the standard of reference was represented by a 3-year follow-up with MRI and/or mammography and/or digital breast tomosynthesis and/or ultrasound. Imaging follow-up was established as the most reasonable standard of reference for this subset of lesions, given the impossibility of obtaining a histological sample [15].

### MRI protocol

MRI examinations were performed on a 1.5-T magnet (Magnetom Aera, Siemens Healthineers) using a bilateral 16-channel coil. The acquisition parameters are reported in Table 1. The reported time of echo (TE) was set at the minimum possible, associated with a receiver bandwidth of 1602 Hz/pixel, an Echoplanar imaging factor of 102, and an echo spacing of 0.77 ms. The diffusion gradients were oriented along the three main space directions using the “3 scan trace modality”. The ADC map was generated by the vendor’s software (Leonardo Syngo.via, Siemens Healthineers), with ADC values calculated as ADC = ln(signal at b 0/signal at b 800)/(b800-b0). After DWI, dynamic contrast-enhanced imaging (DCE) was performed with intravenous administration of 0.1 mmol/kg of gadoteridol (Prohance, Bracco Imaging) at an injection rate of 2 ml/s.

**Table 1:** Acquisition protocol of breast magnetic resonance imaging. The diffusion-weighted imaging (DWI) sequence was acquired before contrast administration. Image subtraction was applied to all post-contrast images

	DWI	DCE	T2-weighted imaging
Sequence	Single-shot EPI	3D FLASH	TSE
Plane	Transverse	Transverse	Transverse
TR/TE (msec)	5700/60	9/4.76	4900/76
Field of view (mm x mm)	350 × 171.6	350 × 350	340 × 340
Matrix (pixels x pixels)	102 × 208	492 × 512	384 × 384
Slice thickness (mm)	4	2	3
Interslice gap (mm)	1	-	0.8
In-plane resolution (mm)	1.7 × 1.7	0.7 × 0.7	0.9 × 0.9
Number of slices	32	80	43
b-values (s/mm^2^)	0, 800	-	-
Number of excitations	2 for b = 0, 4 for b = 800	1	2
Fat saturation	SPAIR	None	None
Parallel imaging (algorithm/acceleration factor)	GRAPPA/2	GRAPPA/2	GRAPPA/2
Acquisition time	1.43 min	1 unenhanced phase followed by 5 post-contrast phases, 1.32 min each	4.41

*DCE* dynamic contrast-enhanced imaging, *EPI* echoplanar imaging, *FLASH* fast long angle shot, *GRAPPA* GeneRalized Autocalibrating Partial Parallel Acquisition, *TE* echo time, *TSE* turbo spin echo, *TR* repetition time, *SPAIR* spectrally adiabatic inversion recovery

### Image analysis

A study coordinator organized independent reading sessions involving two radiologists with high (> 20 years; reader 1 [R1]) and low experience (< 2 years; reader 2 [R2]) in breast imaging, blinded to clinical, histopathological, or follow-up information. The study readings were preceded by a 1-week period of reading sessions in which R1 and R2 were recalled with the rules of ADC assessment established in the EUSOBI document [7] to achieve consistent measurements.

During each session, the coordinator initially showed readers T2-weighted imaging (T2WI) and DCE images only, asking them to identify any relevant finding (no upper limits in number) and categorize each of them according to the BI-RADS, fifth edition [6] (reading phase 1). Subsequently, the coordinator disclosed all the DWI images and the ADC map to apply EUSOBI criteria (reading phase 2) [7]. In particular, readers were asked to report any visible correlation of lesions found on DCE or T2WI imaging on *b* = 800 s/mm^2^ images. Second, they measured the ADC of lesions ≥ 6 mm in size, placing a circular region of interest (ROI) over the most hypointense region of the lesion on the ADC map, paying attention to avoid artifacts, hemorrhage, or necrosis, as well as checking that the region fell within the enhancing part of the lesion on DCE and hyperintense part on the *b* = 800 s/mm^2^ image to avoid the potential confounder of the blackout effect. Readers were allowed to adapt this strategy by placing a ROI over the whole lesion in the case of smaller observations.

In reading phase 3, R1 and R2 were left free to evaluate the MRI information as a whole, i.e., by adding DLs to DCE and T2WI in image interpretation. Readers were asked to assume that the DL of the lesion corresponded to the EUSOBI reference one within which the measured ADC lay and were left free to consider the ADC value relevant or not based on the per-patient combination of DCE and T2WI. Reference DLs were [7] “very low” for ADC values ≤ 0.9 × 10^–3^ mm^2^/s, “low” for ADC values 0.91–1.3 × 10^–3^ mm^2^/s, “intermediate” for ADC values 1.31–1.7 × 10^–3^ mm^2^/s, “high” for ADC values 1.71–2.1 × 10^–3^ mm^2^/s, and “very high” for ADC values > 2.1 × 10^–3^ mm^2^/s. Based on subjective interpretation, the comprehensive risk of malignancy of MRI findings was expressed with a Likert scale, as follows: 1 = highly unlikely; 2 = unlikely; 3 = indeterminate; 4 = likely; 5 = very likely. When the ADC was not measurable, e.g., because of no clear lesion on the ADC map, readers were asked to make the Likert category correspondent to the BI-RADS one.

### Data analysis

ADC values were reported with median values with the interquartile range (IQR) as they were not normally distributed according to the Shapiro–Wilk test. However, we also reported mean values with ± standard deviation (SD) to facilitate the comparison with previous literature. The ADC values measured by R1 and R2 were compared with the Wilcoxon signed-rank test, while the differences between benign and malignant lesions were assessed with the u Mann–Whitney test. The inter-reader agreement in measurements was assessed with Bland–Altman analysis and the intraclass correlation coefficient (ICC). The results of the Bland–Altman analysis were expressed as dimensionless values given a preliminary logarithmic transformation of the data. ICC reference values were as follows [16]: 0.40 = poor; 0.40–0.59 = fair; 0.60–0.74 = good; 0.75–1.00 = excellent. ICC was also used to quantify the inter-reader agreement in assigning DLs corresponding to the measured ADC.

The diagnostic accuracy in assessing malignancy was evaluated with the receiver operating characteristics (ROC) analysis and expressed with an area under the curve (AUC). The sensitivity and specificity were calculated in correspondence with the cut-off of the BI-RADS category or Likert category with the highest Youden’s index. The AUCs were compared with the DeLong test. We did not run the ROC analysis concerning DLs because not all lesions showed measurable ADC. We then calculated the malignancy rate on a per-DL basis, defined as the percent ratio between the total number of cancers found and the total number of lesions assigned to a certain DL.

Analysis was performed with commercially available software (MedCalc Software version 19.8 Ltd).

## Results

### Histological and MRI features

Histological characteristics of 181/201 breast lesions referred to biopsy or surgery are summarized in Table 2. The remaining 20/201 findings included miscellaneous lesions categorized as benign on MRI, showing no evolution (*n* = 15) or disappearance (*n* = 5) during imaging follow-up. Overall, the prevalence of malignant lesions was 67.7% (136/201) (95%CI: 60.7–74.1). Nine high-risk B3 lesions were included among benign lesions after the results of excisional biopsy.

**Table 2:** Histological characteristics of 181/201 breast lesions referred to biopsy or surgery. Percentage values were calculated over the total number of breast lesions (*n* = 201). See the main text for details on the remaining 20/201 lesions referred to follow-up

	Histological type		Number	Prevalence % (95%CI)	Grade (number of cases)
*Malignant lesions* *(n* = *136)*	IDC		86	42.7 (36–50)	G1 (10), G2 (35), G3 (37)
	DCIS		20	9.9 (5–14)	G1 (5), G2 (4), G3 (11)
	ILC		21	10.4 (5–14)	G2 (18), G3 (3)
	Other cancers	IDC and DCIS	1	-	G3
		DCIS and invasive mucinous carcinoma	1	-	G2
		IDC-L	3	-	G2
		Mucinous carcinoma	1	-	G2
		Invasive micropapillary carcinoma	1	-	G3
		Intraductal papillary carcinoma	1	-	G1
		Apocrine carcinoma	1	-	G2
		TOTAL of “Other cancers”	9	4.5 (1–7)	-
*Benign lesions* *(n* = *45)*	Fibroadenoma		24	11.9 (7–16)	-
	Fibrocystic disease		10	4.9 (2–8)	-
	Other benign lesions	Intraductal papilloma	2	-	-
		Sclerosing adenosis	2	-	-
		Benign phylloid tumor	1	-	-
		Atypical lobular hyperplasia	2	-	-
		Usual ductal hyperplasia	2	-	-
		Atypical ductal hyperplasia	2	-	-
		TOTAL of “Other benign leisons”	11	5.5 (2–8)	

*IDC* invasive ductal carcinoma, *DCIS* ductal carcinoma in situ, *ILC* invasive lobular carcinoma, *IDC-L* invasive ductal and lobular carcinoma

On MRI, lesions appeared as masses or non-mass enhancement in 153/201 (76.1%; 95%CI: 69.6–81.8) and 48/201 cases (23.8%; 95%CI: 18.2–30.4), respectively. The mean lesion size was 23 ± 18 mm (range 6–80 mm).

### ADC values and inter-reader agreement

**Fig. 2:**
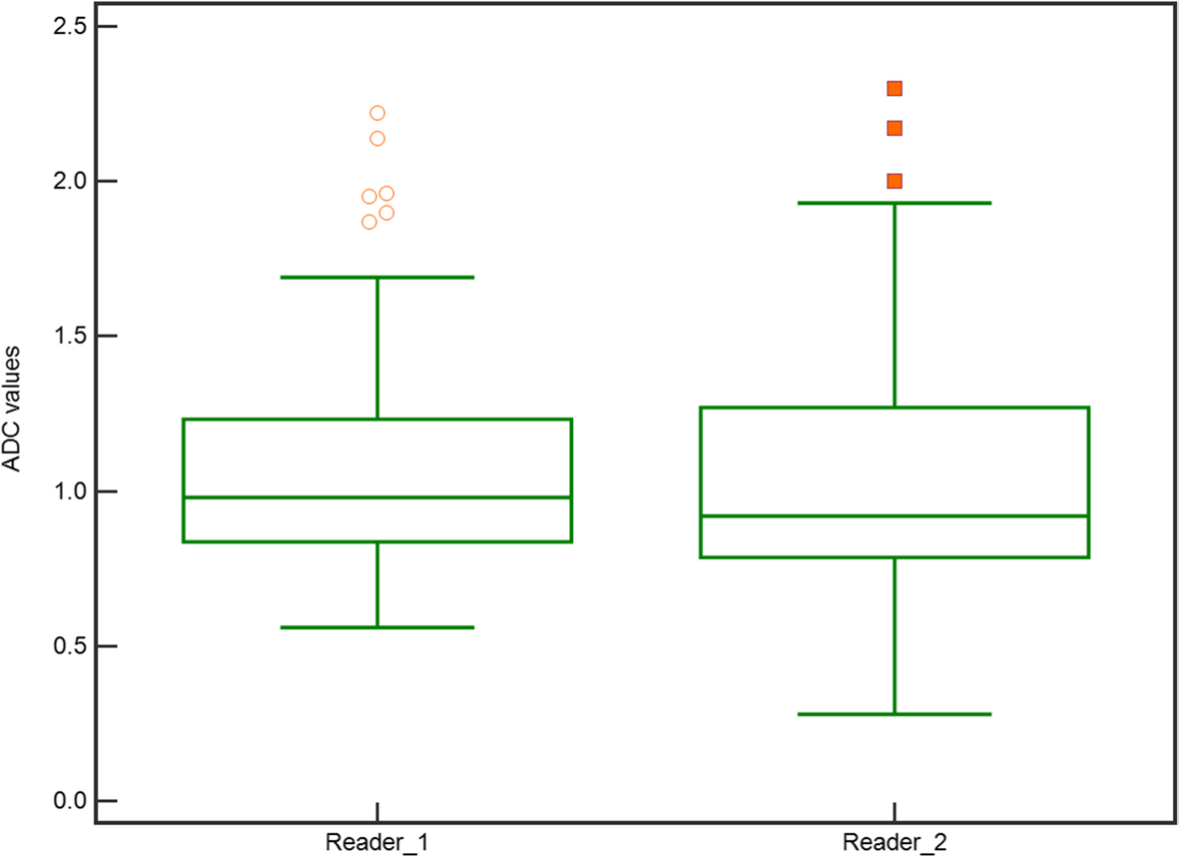
Box and whiskers plot showing the distribution of the apparent diffusion coefficient (ADC) values of breast lesions as measured by reader 1 and reader 2. ADC values on the y-axis are expressed as × 10^–3^ mm^2^/s

Lesion ADC was measurable in 127/201 cases for R1 (63.2%) and 134/201 cases for R2 (66.7%), with median (IQR)/mean ± SD values of 0.98 (0.83–1.26)/1.08 ± 0.35 and 0.92 (0.78–1.3)/1.04 ± 0.36 × 10^–3^ mm^2^/s, respectively (Fig. 2) (*p* = 0.0058). The ADC was significantly lower in malignant than benign lesions (*p* < 0.001) for both R1 (median [IQR]/mean ± SD 0.90 [0.78–1.02]/0.91 ± 0.22 versus 1.50 [1.25–1.62]/1.44 ± 0.31 × 10^–3^ mm^2^/s) and R2 (median [IQR]/mean ± SD 0.85 [0.75–0.96]/0.87 ± 0.24 versus 1.46 [1.27–1.58]/1.42 ± 0.31 × 10^–3^ mm^2^/s).

**Fig. 3:**
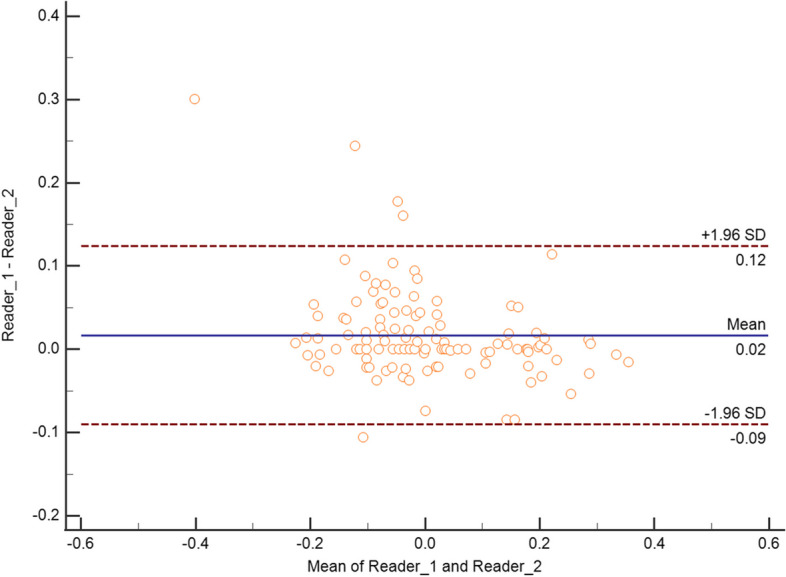
Bland–Altman graph plotting the mean (x-axis) versus the difference (y-axis) of any paired measurement of ADC values by reader 1 and reader 2, including the mean absolute difference (blue line) and 95% limits of agreement (dashed lines). ADC values are expressed as dimensionless values after logarithmic transformation

**Fig. 4:**
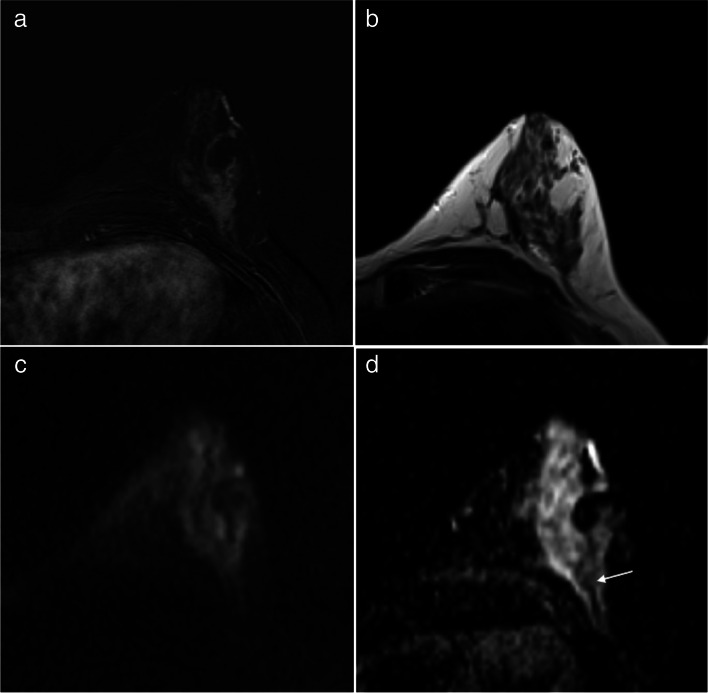
True-positive assessment of a ductal carcinoma in situ associated with invasive ductal carcinoma in a 46-year-old woman. The hypervascular left-sided lesion showed both mass and non-mass components on the first subtracted post-contrast image (**a**) while appearing relatively isointense to the fibroglandular background on T2-weighted imaging (**b**). The lesion signal was inhomogeneous on the high b-value image (**c**), with a markedly hyperintense area corresponding to contrast enhancement and a zone appearing as markedly hypointense compared to the surrounding tissue on the ADC map (arrow in **d**). Despite the inhomogeneous lesion appearance, both readers independently selected this zone for placing the ROI and obtained comparable ADC values of 0.75 e 0.72 × 10^–3^ mm^2^/s. The lesion was classified BI-RADS 5 and Likert 5 by both

The mean difference in ADC values measured by R1 and R2 was minimal on Bland–Altman analysis, as R1 measured ADC values 0.02 times (i.e., 2%) higher than R2 on average. This corresponded to close limits of agreement, i.e., expected differences in R2 measurements ranging from -9% to + 0.12% compared to what measured by R1 (Fig. 3). The inter-reader agreement was excellent in measuring ADC (ICC = 0.948 [95%CI 0.925–0.965]) and assessing DLs (ICC = 0.989 [95%CI 0.988–0.991]) (Fig. 4). Table 3 shows the malignancy rate on a per-DL basis.

**Table 3:** Distribution of breast lesions across different diffusion levels and related malignancy rate on a per-diffusion level

	Reader 1				Reader 2			
Diffusion level	Number of lesions	% (95%CI)	Malignancy rate	% (95%CI)	Number of lesions	% (95%CI)	Malignancy rate	% (95%CI)
Very low	50/201	24.9 (18.9–30.9)	48/50	96.0 (90.6–100)	63/201	31.3 (24.9–37.7)	61/63	96.8 (92.4–100)
Low	49/201	24.4 (18.5–30.3)	37/49	75.5 (63.5–87.5)	42/201	20.9 (15.3–26.5)	30/42	71.4 (57.7–85.1)
Intermediate	21/201	10.4 (6.18–14.6)	2/21	9.5 (1.0–22)	22/201	10.9 (6.8–16.5)	2/22	9.1% (1.1–32.8)
High	5/201	2.5 (0.8–5.8)	0/5	0 (–)	5/201	2.5 (0.8–5.8)	0/5	0 (–)
Very high	2/201	1 (0.01–2.4)	1/2	0.5 (19–100)	2/201	1 (0.01–2.4)	1/2	0.5 (19–100)
Diffusion level not attributable (non-measurable ADC)	74/201	36.8 (28.9–46.2)	48/74	64.8 (47.8–86)	67/201	33.3 (26.8–39.8)	42/67	62.7 (51.1–74.3)

*ADC* apparent diffusion coefficient

### Diagnostic accuracy

**Fig. 5:**
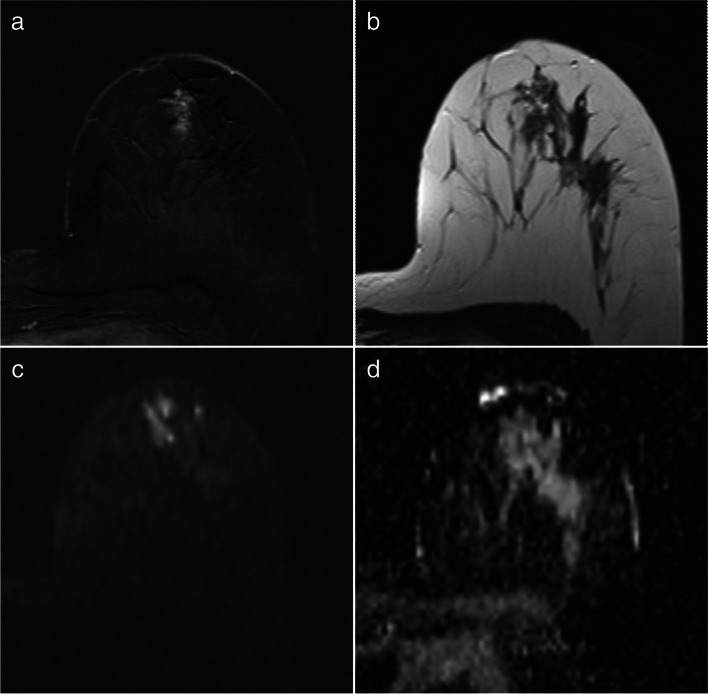
False-positive case avoided by Likert categorization in a 49-year-old woman with a histological diagnosis of fibrocystic disease. The left-sided breast lesion presented as a non-mass enhancement area on the first-subtracted post-contrast sequence (**a**), with isointensity compared to the fibroglandular tissue on T2-weighted imaging (**b**). On Diffusion-weighted imaging, the whole lesion appeared hyperintense on both the high b-value image (**c**) and ADC map (**d**). Reader 1 and reader 2 measured an ADC value of 1.90 and 1.46 × 10^–3^ mm.^2^/s, respectively, and downgraded the level of suspicion from BI-RADS 4 to Likert 2 (reader 1) and from BI-RADS 4 to Likert 3 (reader 2)

Table 4 summarizes which lesions were reclassified using Likert categorization, and whether reclassification was correct compared to the standard of reference (Fig. 5). Notably, most reclassifications concerned the same cases for both readers. As an overall balance, Likert categorization saved 2 false-positives for both R1 and R2, and 2 false-negatives and 1 false-negative for R1 and R2, respectively. While Likert categorization induced false-positives and avoided false-negatives in lesions with a “low” DL, saved false-positives cases showed lesion ADCs in the “low-to-intermediate” DL (Table 4). The single false-negative case induced by Likert categorization for R2 showed a “low” DL.

**Table 4:** Characteristics of the lesions reclassified as benign (or vice versa) by Likert score after initial BI-RADS-based assessment

		Both readers				One reader only			
		Number of lesions	Mean size (mm) (range)	Pattern on DCE	ADC value (R1/R2) (× 10^–3^ mm^2^/s)	Number of lesions (reader)	Mean size (mm) (range)	Pattern on DCE	ADC value (× 10^–3^ mm^2^/s)
False-positive cases at BI-RADS categorization correctly assessed as benign by Likert categorization (False-positives avoided by Likert categorization)	Fibroadenoma	2	25.5 (19–32)	2 ME	1.60/1.58, 1.25/1.30	-	-	-	-
	Fibrocystic breast disease	1	10 (-)	1 ME	1.90/1.46	1 (R1)	17 (-)	NME	1.40
	Benign lesions disappearance during the follow-up	-	-	-	-	1 (R2)	12 (-)	ME	1.33
True-negative cases at BI-RADS categorization incorrectly assessed as malignant by Likert categorization (False-positives induced by Likert categorization)	Intraductal papilloma	1	16 (-)	ME	1.16/1.24	-	-	-	-
	Sclerosing adenosis	1	23 (-)	NME	1.09/1.07	-	-	-	-
False-negative cases at BI-RADS categorization correctly assessed as malignant by Likert categorization (False-negatives avoided by Likert)	IDC, grade 3	1	11 (-)	NME	1.07/1.07	-	-	-	-
	DCIS, grade 1	1	13 (-)	ME	0.94/0.92	-	-	-	-
True-positives at BI-RADS categorization incorrectly assessed as benign cases by Likert categorization (False-negatives induced by Likert)	IDC, grade 1	-	-	-	-	1 (R2)	10 (-)	ME	0.92

*IDC* invasive ductal carcinoma, *DCIS* ductal carcinoma in situ, *ME* mass enhancement, *NME* non-mass enhancement, *R1* reader 1, *R2* reader 2

**Fig. 6:**
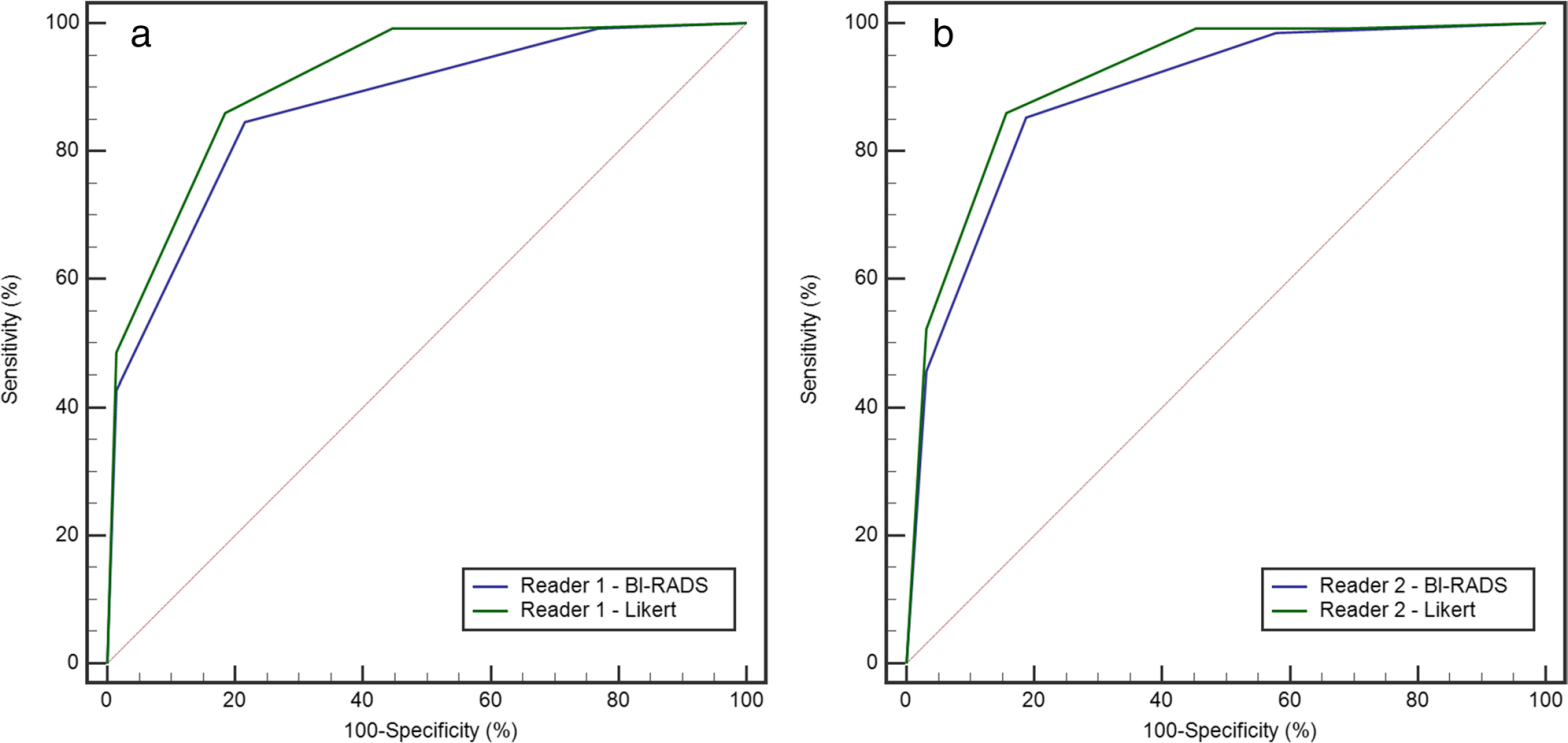
Receiver operating characteristics (ROC) curves for the diagnosis of breast cancer made by reader 1 (**a**) and reader 2 (**b**) using the Breast Imaging Reporting and Data System (BI-RADS) or the Likert score integrating diffusion-weighted imaging

Overall, Likert categorization showed higher AUC (Fig. 6), sensitivity, and specificity than BI-RADS categorization (Table 5). Supplementary Table 1 shows the overview of correct and incorrect lesion classifications compared to the standard of reference, while Supplementary Table 2 reports the cases incorrectly assessed by both BI-RADS and Likert categorization.

**Table 5:** Diagnostic accuracy for malignancy of the Breast Imaging Reporting and Data System (BI-RADS)-based and Likert-based readings (BI-RADS plus diffusion level) of breast magnetic resonance imaging

		Sensitivity (95%CI)	Specificity (95%CI)	AUC (95%CI)	*p*	Cut-off category at ROC analysis
Reader 1	BI-RADS	84.6 (77.4–90.2)	78.5 (66.5–87.7)	0.87 (0.81–0.91)	0.0208	≥ 4
	Likert	86.0 (79.0–91.4)	81.5 (70.0–90.1)	0.91 (0.86–0.95)		≥ 4
Reader 2	BI-RADS	85.3 (78.2–90.8)	81.2 (69.5–89.9)	0.89 (0.83–0.93)	0.1171	≥ 4
	Likert	86.0 (79.0–91.4)	84.4 (73.1–92.2)	0.91 (0.86–0.95)		≥ 4

*AUC* area under the curve at the receiver operating characteristics analysis, *ROC* receiver operating characteristics

## Discussion

We compared breast lesion categorization using the BI-RADS on DCE and T2-weighted imaging versus Likert categorization on the whole examination including DLs [7], showing three main results. First, adding DLs to image interpretation improved the specificity for cancer from 78.5% to 81.5% in the case of R1 and 81.2 to 84.4% in the case of R2, translating into a significant improvement in the AUC for one out of two readers. Second, DLs were found to reliably reflect the risk of malignancy, as the large majority of cancers were associated with lower ADC values, with a malignancy rate in the “very low” and “low” DLs of 96.1% and 80%, respectively. Third, the use of a standardized method of measurement translated into an excellent inter-reader agreement in measuring both the ADC (ICC 0.948) and establishing the DLs (ICC 0.989). According to Bland–Altman analysis, ADC values showed minimum mean differences and close limits of agreement (i.e., minimal expected discrepancies) when measured by R1 and R2. Taken together, our results validate the DLs as a simple and reproducible means to include the quantitative information of the ADC in the interpretation of breast MRI and match the expected task of improving the specificity of the examination [17–19]. This also emphasizes the potential for DLs to reduce unnecessary biopsy procedures [20] and help to increase the use of DWI in clinical practice [7].

Several previous works investigated the diagnostic value of the ADC in assessing the malignancy of breast lesions [2–4, 21, 22]. However, as far as we know, only a recent study by Bickel et al [11] evaluated the potential of DLs to overcome the well-known problem of establishing absolute ADC thresholds in clinical practice. The Authors derived a system of six DLs ranging from 1 (“ADC not measurable”) to 5 (“very low”) from a multicentric population of 1625 women with 1736 pathologically confirmed lesions, showing that at an ADC threshold of < 1.0 × 10^–3^ mm^2^/s distinguishing between the “intermediate/low” from “low” DL, the positive predictive value for malignancy was 95.8%. Comparably to these authors, we found that the ADC values of malignant lesions were lower than benign ones, as expected [8]. Differently from them, we did not derive the DLs from the patient population under analysis but used the predefined ones prompted in an EUSOBI mission and consensus statement [7], which in turn originated from a metanalysis [10]. One might argue that using predefined values carried the risk of testing DLs obtained from a different distribution of histological subtypes of breast cancer compared to the study cohort. However, the added value we observed can be considered reasonable proof that the DLs we used can be successfully and reliably applied in an external cohort, thus being of potential clinical applicability. This is in line with the fact that the dataset of origin was large enough to reasonably adjust for fluctuations of lesions’ distribution and type (61 studies and 5205 breast lesions) [10], and the small differences between the DLs used by us and Bickel et al [11] (≤ 0.9 versus < 1.0 × 10^–3^ mm^2^/s for the “very low” level, 0.91 to 1.7 versus 1.0 to < 1.5 × 10^–3^ mm^2^/s for the “low-to-intermediate” level, 1.71–2.1 versus 1.5 to < 1.9 × 10^–3^ mm^2^/s for the “high” level, and > 2.1 versus ≥ 1.9 × 10^–3^ mm^2^/s for the “very high” level).

We believe there are two points of strength in our work. First, unlike the above Authors [11], we did not evaluate DLs as a stand-alone diagnostic tool but investigated the effect of adding them to breast MRI. We also found that there was no price to pay for increased specificity as the use of DLs was associated with a slight but measurable increase in sensitivity as well, which could be of special benefit in the preoperative setting. However, larger studies should investigate whether the overall balance between sensitivity and specificity favors the use of DWI in a certain clinical scenario. Second, we observed that ADC could not be provided in around 37% of lesions for both R1 and R2, thus making DLs unavailable. Rather than being a limitation, this result emphasizes that DWI is a complementary tool to the anatomical and functional information deriving from DCE and T2-weigthed imaging and that lesion characterization cannot be made upon DLs alone [7]. However, we acknowledge that further technical development in DWI sequences could improve our results by making more lesions measurable on the ADC map, e.g., because of increased spatial resolution. This line of research should be considered of primary importance to enhance the role of DWI in breast imaging.

This study is not devoid of limitations. Likert categorization was subjective, i.e., we did not provide predefined rules for the combined interpretation of DCE, T2WI, and DLs, e.g., as occurs with the Prostate Imaging Reporting and Data System (PI-RADS) [23]. Likert categorization induced false-positives and avoided false-negatives in lesions with a “low” DL (rate of malignancy 71.4–75.5% in our series), suggesting that readers trusted considerably the low ADC value as a problem-solving tool, despite this carries the risk of translating into errors. On the contrary, we do not have a definite explanation of why saved false-positives and one single induced false-negative at Likert categorization mainly showed “intermediate” DLs (rate of malignancy 9.1–9.5%) or even a “low” DLs of 0.92 10^–3^ mm^2^/s. One can hypothesize that, for reasons difficult to assess on a case-by-case basis, readers’ interpretation was influenced more by T2-weighted imaging, DCE, or even high b-values images than the ADC when making the overall assessment of these lesions. This emphasizes the need for further studies exploring how to weigh and combine the ADC value and individual MRI sequences into final lesion categorization. Second, while differing in experience in breast imaging, both readers came from the same institution, so the consistency we observed in Likert categorization could have been affected by comparable interpretation criteria. Finally, we did not investigate additional well-established tasks for DWI, such as differentiating between invasive versus noninvasive lesions [21, 24–27].

In conclusion, using predefined DLs prompted by EUSOBI in a mission and consensus statement for expanding the use of DWI in breast MRI, we observed that higher malignancy rates were associated with “very low” and “low” levels, as expected. Integrating DLs into the diagnostic process improved both the sensitivity and specificity for breast cancer compared to the BI-RADS-based interpretation of DCE and T2-weighted imaging alone, even using a subjective Likert scale. The improvement was greater for specificity, suggesting that, when measurable, DLs can avoid unnecessary biopsy recommendations as the main effect.

### Supplementary Information

Below is the link to the electronic supplementary material.

Supplementary file1 (PDF 50 KB)

## Abbreviations

ADC: Apparent diffusion coefficient
BI-RADS: Breast imaging reporting and data system
CI: Confidence interval
DCE: Dynamic contrast-enhanced imaging
DL: Diffusion level
DWI: Diffusion-weighted imaging
EUSOBI: European Society of Breast Imaging
IQR: Interquartile range
MRI: Magnetic resonance imaging
R1: Reader 1
R2: Reader 2
ROI: Region of interest
SD: Standard deviation

## Declarations

### Guarantor

The scientific guarantor of this publication is Rossano Girometti.

### Statistics and biometry

Rossano Girometti has significant statistical expertise and performed statistical analysis. No advanced statistical methods were necessary for this paper.

### Study subjects or cohorts overlap

Study subjects have not been previously reported in other works.

### Methodology

• retrospective

• cross-sectional, observational study

• performed at one institution
