# Supplementary material for: Added value of the EUSOBI diffusion levels in breast MRI
Source: Eur Radiol. 2023 Nov 7;34(5):3352–63. doi: 10.1007/s00330-023-10418-4 (PMC11126436; doi:10.1007/s00330-023-10418-4)
Supplement: Supplementary file 1 — Supplementary file1 (PDF 50 KB) [file 330_2023_10418_MOESM1_ESM.pdf]

Added value of the EUSOBI diffusion levels in breast MRI

Electronic Supplementary Material

Supplementary Table 1 - Distribution of the true positive, false negative, false positive, and true negative assessment of breast lesions according to the BI-RADS and Likert categorization.

			LIKERT				
			TP	FN	TP	TN	
**Reader 1**	**BI-RADS**	TP	115	0	-	-	(115)
		FN	2	19	-	-	(21)
		FP	-	-	10	4	(14)
		TN	-	-	2	49	(51)
			(117)	(19)	(12)	(53)	
**Reader 2**		TP	115	1	-	-	(116)
		FN	2	18	-	-	(20)
		FP	-	-	8	4	(12)
		TN	-	-	2	51	(53)
			(117)	(19)	(10)	(55)	

FN = false-negative; FP = false-positive; TN = true-negative; TP = true-positive.

Supplementary Table 2 - Characteristics of the cases corresponding to false-negative and false-positive cases using both the BI-RADS and Likert categorization.

	Reader 1			Reader 2		
**False-negative cases**	Number (grade)	Mean size (range) (mm)	Pattern on DCE	Number (grade)	Mean size (range) (mm)	Pattern on DCE
DCIS	5 (4 grade 1, 1 grade 2)	10.8 (6-15)	3 ME, 2 NME	4 (4 grade 1)	11.2 (6-15)	3 ME, 1 NME
IDC	10 (2 grade 1, 5 grade 2, 2 grade 3)	16.8 (6-40)	5 ME, 5 NME	10 (4 grade 1, 3 grade 2, 2 grade 3)	13.9 (6-40)	4 ME, 6 NME
ILC	4 (4 grade 2)	24 (7-70)	3 ME, 1 NME	5 (5 grade 2)	25.2 (7-70)	3 ME, 2 NME
**False-positive cases**						
Fibrocystic disease	3	39.3 (14-64)	1 ME, 2 NME	3	39.3 (14-64)	1 ME, 2NME
Atypical lobular hyperplasia	1	19 (-)	NME	1	19 (-)	NME
Atypical ductal hyperplasia	2	22 (9-35)	1 ME, 1 NME	1	35 (-)	NME
Usual ductal hyperplasia	1	23 (-)	ME	1	23 (-)	ME
Benign phylloid tumor	1	20 (-)	NME	1	20 (-)	NME
no evolution during imaging follow-up	2	10 (8-12)	2 ME	1	8 (-)	ME

IDC = invasive ductal carcinoma; DCIS = ductal carcinoma in situ; ILC = invasive lobular carcinoma; ME = mass enhancement; NME = non-mass enhancement.
